# Respiratory adverse events associated with PD-1/PD-L1 inhibitors: an analysis based on the FDA adverse event reporting system

**DOI:** 10.3389/fonc.2026.1788532

**Published:** 2026-04-10

**Authors:** Zhenyu Wang, Shuting Cui, Guimei Wang, Shanting Qiu, Jianan Jin, Hanliang Jiang

**Affiliations:** 1 Pulmonary and Critical Care Medicine, Regional Medical Center for National Institute of Respiratory Disease, Sir Run Run Shaw Hospital, School of Medicine, Zhejiang University, Hangzhou, Zhejiang, China; 2 School of Medicine, Shaoxing University, Shaoxing, Zhejiang, China; 3 Graduate School, Zhejiang Chinese Medical University, Hangzhou, Zhejiang, China

**Keywords:** adverse events, FAERS, immune checkpoint inhibitors, pharmacovigilance, respiratory system

## Abstract

**Background:**

Respiratory adverse events associated with programmed cell death protein 1 and programmed death-ligand 1 (PD-1/PD-L1) inhibitors have emerged as an important safety concern in clinical oncology. With the expanding use of immune checkpoint inhibitors (ICIs), a comprehensive evaluation of real-world respiratory toxicity is essential to optimize clinical risk management. This study aimed to systematically characterize the occurrence patterns and pharmacovigilance signals of respiratory adverse events related to PD-1/PD-L1 inhibitors using the FDA Adverse Event Reporting System (FAERS).

**Methods:**

Reports of suspected adverse events associated with PD-1/PD-L1 inhibitors were extracted from the FAERS database, covering the period from the first quarter of 2004 to the second quarter of 2025. Respiratory, thoracic, and mediastinal disorders were identified according to the System Organ Class (SOCs) and Preferred Terms (PTs) of MedDRA version 27.0. Disproportionality analyses were performed using reporting odds ratio (ROR), proportional reporting ratio (PRR), information component (IC025), and empirical Bayesian geometric mean (EBGM). Univariable and multivariable logistic regression analyses were further conducted to explore factors associated with respiratory adverse event reporting.

**Results:**

A total of 163,193 reports identified PD-1/PD-L1 inhibitors as the primary suspected drugs. Pneumonia-related signals were consistently detected across most ICIs, indicating a shared pattern of pulmonary toxicity encompassing both infectious pneumonia and immune-related pneumonitis. Respiratory adverse events predominantly occurred during the early treatment phase, particularly within the first 60 days, with a secondary increase observed after prolonged exposure exceeding six months. Elderly patients (≥65 years) accounted for a substantial proportion of cases and exhibited a higher frequency of fatal outcomes. Regression analyses demonstrated that sex, reporting region, and drug class were significantly associated with respiratory adverse event reporting.

**Conclusions:**

PD-1/PD-L1 inhibitors are associated with a significant risk of respiratory adverse events in real-world clinical practice. Early and targeted respiratory monitoring is warranted, particularly during the initial treatment phase and among elderly patients. These findings highlight the need for optimized risk stratification and proactive management strategies to improve the safety of PD-1/PD-L1 inhibitor therapy.

## 1 Introduction

Immune checkpoint inhibitors (ICIs) have become a major therapeutic advance in oncology over the past decade, markedly reshaping the management of multiple malignancies (1, 2). Agents targeting programmed cell death protein 1 (PD-1) and its ligand PD-L1 act by interrupting the PD-1/PD-L1 interaction, thereby reversing tumor-induced suppression of T-cell activity. This immune modulation restores the effector function of cytotoxic T lymphocytes, facilitates immune-mediated tumor cell clearance, and contributes to more sustained antitumor responses (3, 4). Clinical benefit has been demonstrated across a broad range of solid tumors, including non–small cell lung cancer, metastatic melanoma, renal cell carcinoma, and bladder cancer. As clinical use has expanded, an increasing spectrum of adverse events associated with ICIs has been recognized. Immune-related adverse events (irAEs), defined as a unique spectrum of toxicities arising from the immune system’s cross-reactivity against normal self-tissues during immune checkpoint inhibitor (ICI) therapy, can involve almost any organ.such as the respiratory, endocrine, gastrointestinal, and nervous systems (5). These toxicities are generally attributed to dysregulated immune activation and loss of immune tolerance, and severe manifestations may lead to significant organ dysfunction or even death (6, 7). While irAEs affecting the skin, blood, endocrine organs, and gastrointestinal tract have been extensively studied (8–11), respiratory adverse events remain comparatively less well characterized. Given their appreciable occurrence in routine practice and their potential for fatal outcomes, further investigation and accumulation of real-world evidence in this area are needed.

The FAERS is one of the largest global databases for adverse event reporting and represents a key resource for post-marketing safety surveillance and pharmacovigilance research. FAERS is based on voluntarily submitted reports and includes multidimensional information on drug exposure, patient characteristics, and clinical outcomes. This structure supports the application of disproportionality-based signal detection methods, including reporting odds ratios (ROR), proportional reporting ratios (PRR), and information components (IC), to assess potential AE detection (12). Accordingly, real-world analyses using FAERS provide an effective framework for identifying safety signals related to PD-1/PD-L1 inhibitors and for exploring their occurrence patterns, temporal distributions, and risk heterogeneity.

Given the potential safety concerns associated with PD-1/PD-L1 inhibitors, updated real-world analyses are needed to better delineate their relationship with respiratory adverse events and to identify relevant influencing factors. Previous studies using FAERS or VigiBase have explored this topic, establishing a foundational link between PD-1/PD-L1 inhibitors and respiratory toxicities. For instance, an analysis of FAERS data (2014-2021) identified significant associations with interstitial lung disease (ILD) and pneumonitis, noting that 75.40% of events occurred within the first three months of treatment (13). Similarly, research using VigiBase (through 2019) confirmed that these agents are frequently associated with serious or fatal events, including pulmonary embolism and respiratory failure (14). However, significant knowledge gaps remain. Most existing literature utilizes data only up to 2021, missing the rapid expansion of ICI utilization and the clinical introduction of newer inhibitors in the last three years. As the treated population and exposure scale have changed markedly, updated real-world analyses are required to delineate current safety patterns.

According to the latest raw statistics and our preliminary calculations from the FDA FAERS public dashboard (15) in the first half of 2025 alone, newly reported adverse events related to pembrolizumab exceeded 5,000 cases, representing approximately 10.42% of its cumulative reports. Reports involving toripalimab retrieved from the same official source have also increased rapidly, rising from 61 cases in 2023 to 111 in 2024 and 637 in 2025, corresponding to year-on-year growth rates of 81.97% and 473.87%, respectively.

In this context, a re-examination of the epidemiological features, temporal patterns, and clinical outcome differences of adverse events associated with PD-1/PD-L1 inhibitors is needed. The present study therefore established a systematic pharmacovigilance analysis framework using the most recent FAERS data. By combining disproportionality-based signal detection with complementary analytical approaches, we aimed to describe the occurrence patterns, risk trends, and possible mechanisms of the most frequent respiratory AEs, encompassing both general symptoms and specific pulmonary immune-related adverse events (pirAEs) related to PD-1/PD-L1 inhibitors, with the goal of informing safety monitoring and risk management in clinical practice.

## 2 Materials and methods

### 2.1 Data source

**Figure 1:**
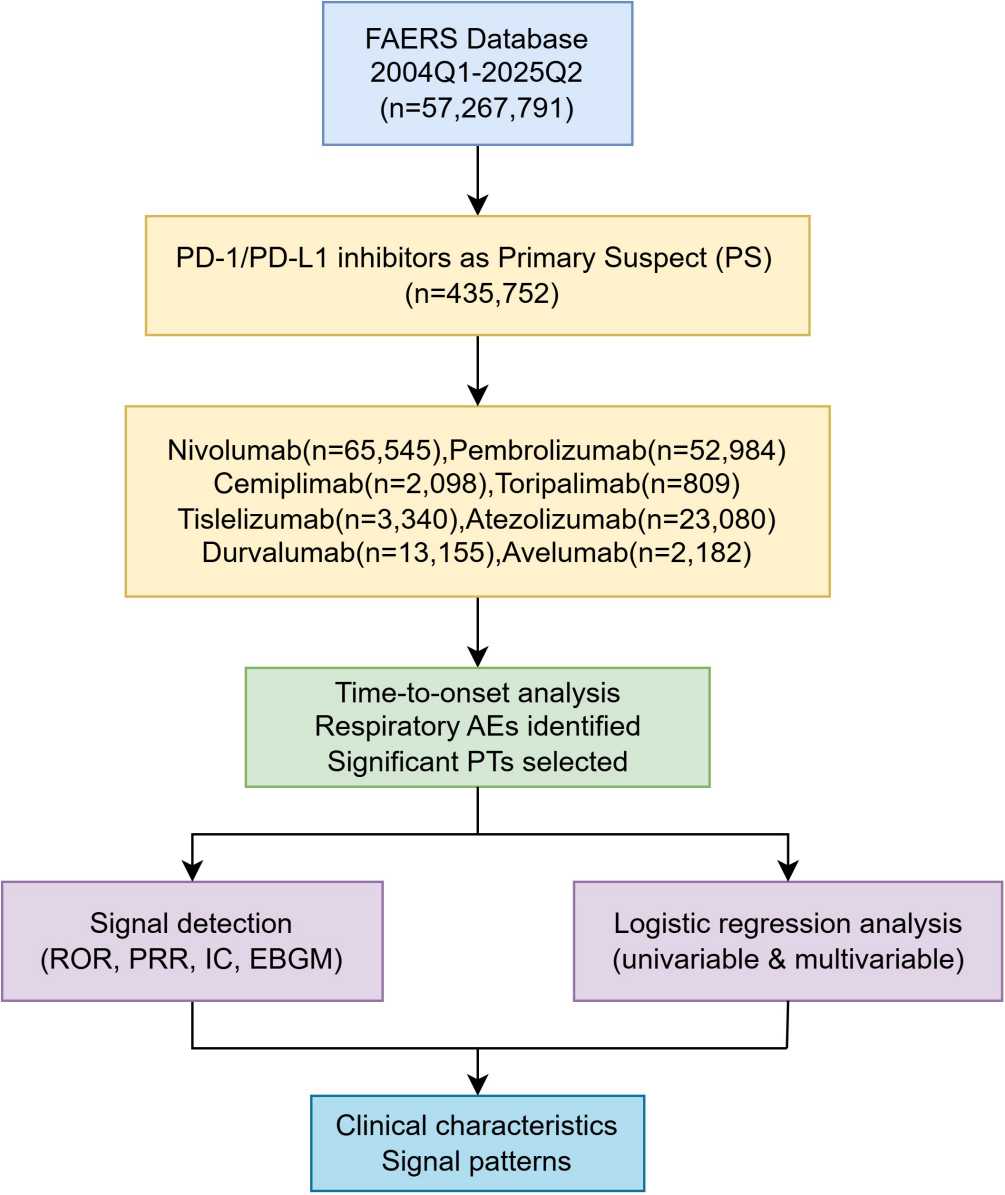
Flowchart.

This study was based on the FAERS, including all adverse event (AE) reports submitted from the first quarter of 2004 to the second quarter of 2025. FAERS currently contains over 20 million reports worldwide, submitted by both healthcare professionals (e.g., physicians and pharmacists) and non–healthcare reporters, such as patients, consumers, and legal representatives. To ensure the specificity of the identified signals and enhance potential causal relevance, we restricted our inclusion to reports where PD-1/PD-L1 inhibitors were identified as the ‘Primary Suspected (PS)’ drug. This methodological choice is designed to minimize confounding influences from concomitant medications, allowing for a clearer evaluation of the direct association between these specific immunotherapy agents and respiratory adverse events. The included agents represent the currently mainstream inhibitors widely used in clinical practice, with detailed regulatory and pharmacological profiles available via the Drugs@FDA database. Each report is assigned a unique case identifier and includes information on patient demographics, reporting time and country, reporter type, suspected drugs and indications, time to event onset, seriousness classification, and narrative descriptions of adverse events. The seriousness of adverse events was classified according to the FDA’s regulatory reporting standards. A respiratory AE was defined as ‘serious’ if it resulted in any of the following outcomes: death, life-threatening conditions, initial or prolonged hospitalization, disability or permanent damage, congenital anomalies, or other clinically significant medical events (16). All AEs were standardized using the Medical Dictionary for Regulatory Activities (MedDRA), version 27.0. PTs were used to define individual adverse events, and SOCs were applied for organ-system–level categorization. The analysis focused on AEs classified under “Respiratory, thoracic and mediastinal disorders.” Reports listing PD-1 or PD-L1 inhibitors as the primary suspect (PS) drugs were included, Cases in which these inhibitors were listed as secondary suspect, concomitant, or interacting drugs were excluded to ensure the specificity of our findings. encompassing agents such as nivolumab, pembrolizumab, atezolizumab, durvalumab, and avelumab. To improve data quality, reports were deduplicated and standardized according to FDA-recommended data-cleaning procedures (17). The overall study design and pharmacovigilance workflow are illustrated in **Figure 1**. Reports unrelated to drug exposure, including those involving medical devices, procedural complications, or social and psychological conditions, were excluded.

Notably, due to the inherent one-to-many mapping structure of the FAERS database, a single unique patient case may involve multiple adverse events and designate multiple agents as primary suspected drugs. This is determined by the intrinsic architecture of the database; therefore, the total number of extracted primary suspected drug-event records inevitably exceeds the total number of unique individual cases.

### 2.2 Signal detection and statistical analysis

After data cleaning and deduplication, all adverse event reports related to PD-1/PD-L1 immune checkpoint inhibitors (ICIs) were included, from which respiratory-related PTs were identified. Based on the pooled data across all evaluated PD-1/PD-L1 inhibitors, the eight most frequently reported respiratory adverse events—dyspnoea, pneumonia, pneumonitis, interstitial lung disease, cough, pleural effusion, respiratory failure, and immune-mediated lung disease—were selected to establish a common baseline for subsequent analyses. This approach allowed us to evaluate a broad spectrum of respiratory toxicities, with classic pulmonary irAEs (pirAEs), such as pneumonitis and interstitial lung disease, analyzed as a key component of this study. Furthermore, while the analysis primarily focused on these eight core PTs, additional highly prominent and clinically significant signals detected separately for specific agents (e.g., radiation pneumonitis for durvalumab and pulmonary embolism for avelumab) were supplemented. This approach resulted in 9 to 11 PTs being analyzed for certain drugs, allowing for a more comprehensive and drug-specific safety characterization. Time-to-onset (TTO) was defined as the interval (in days) between the start of ICI therapy and the onset of the adverse event. Reports with missing or illogical dates were excluded. The TTO data were analyzed using descriptive statistics, including percentages across predefined time intervals, without formal comparative testing.

To identify potential safety signals, drawing on numerous previous studies utilizing the FAERS database, these disproportionality-based pharmacovigilance methods were applied to evaluate the strength of the statistical association by comparing the observed frequency of specific drug-adverse event combinations with their expected frequency across the entire database (18). Specifically, four commonly used algorithms were implemented:

1. Reporting Odds Ratio (ROR) was used to assess the strength of the association between a specific drug and a given adverse event; a positive signal was defined when the lower bound of the 95% confidence interval of the ROR (ROR_025_) exceeded 1.
2. Proportional Reporting Ratio (PRR) compared the proportion of a specific adverse event reported for the target drug with that for all other drugs in the database; a signal was considered present when PRR ≥ 2, the chi-square statistic ≥ 4, and the number of reports for the event was at least 3.
3. Information Component (IC), a Bayesian-based signal detection metric, was calculated, with a statistically significant signal defined as a lower 95% credibility interval bound (IC_025_) greater than 0.
4. Empirical Bayesian Geometric Mean (EBGM) was employed to obtain shrinkage estimates and reduce small-sample bias; an abnormal reporting signal was identified when the lower bound of the EBGM credibility interval (EBGM_05_) exceeded 2.

To enhance the robustness and specificity of signal detection, only adverse events simultaneously meeting the positivity criteria of all four methods were considered true safety signals. All data processing and statistical analyses were performed using Microsoft Excel (Office 365) and R software (version 4.4.2). R packages including dplyr, ggplot2, and epiR were used for data cleaning, signal calculation, and visualization.

### 2.3 Univariable and multivariable regression analysis

Adverse event data from eight ICIs—nivolumab, pembrolizumab, cemiplimab, toripalimab, tislelizumab, atezolizumab, durvalumab, and avelumab—were pooled for regression analyses, with respiratory adverse events defined by PTs as the outcome of interest. Key variables, including age, sex, body weight, reporting country (country_code), and drug class (drug_class), were evaluated for completeness and plausibility. Records with missing values or those outside predefined reasonable ranges were excluded, resulting in a cleaned analytical dataset. Continuous variables, namely age and body weight, were categorized using clinically relevant thresholds to facilitate regression analyses. Note that the age categorization in the multivariable analysis was adjusted from the initial descriptive groupings in **Table 1** to avoid statistical bias caused by sparse data cells in the respiratory adverse event subgroup.

**Table 1:** Patient characteristics.

Characteristic	Nivolumab	Pembrolizumab	Cemiplimab	Toripalimab	Tislelizumab	Atezolizumab	Durvalumab	Avelumab	Total
Total Cases	65545	52984	2098	809	3340	23080	13155	2182	163193
Serious	58368(89.05)	43696(82.47)	1813(86.42)	806(99.63)	3339(99.97)	21891(94.85)	12401(94.27)	1897(86.94)	144211(88.37)
PS	175534	155995	6055	1536	6030	58102	27917	4583	435752
Gender									
Female	19939(30.42)	23890(45.09)	479(22.83)	6(0.74)	1(0.03)	7480(32.41)	2997(22.78)	568(26.03)	55360(33.92)
Male	36946(56.37)	26119(49.30)	676(32.22)	10(1.24)	1(0.03)	12591(54.55)	6064(46.10)	1409(64.57)	83816(51.36)
Missing	8660(13.21)	2975(5.61)	943(44.95)	793(98.02)	3338(99.94)	3009(13.04)	4094(31.12)	205(9.40)	24017(14.72)
Age(years)									
< 18	248(0.38)	94(0.18)	2(0.10)	0(0.00)	2(0.06)	27(0.12)	4(0.03)	2(0.09)	379(0.23)
18-44	3325(5.07)	2534(4.78)	26(1.24)	2(0.25)	67(2.01)	1042(4.51)	233(1.77)	45(2.06)	7274(4.46)
45–64	17407(26.56)	12494(23.58)	188(8.96)	6(0.74)	503(15.06)	6231(27.00)	2841(21.60)	476(21.81)	40146(24.60)
≥65	24987(38.12)	22283(42.06)	453(21.59)	8(0.99)	543(16.26)	10664(46.20)	5112(38.86)	1164(53.35)	64214(39.35)
Not Specified	19578(29.87)	15579(29.40)	1429(68.11)	793(98.02)	2225(66.62)	5116(22.17)	4965(37.74)	495(22.69)	49180(30.14)
Weight(kg)									
N(Missing)	18338(47207)	11299(41685)	53(2045)	10(799)	871(2469)	8507(14573)	3532(9623)	825(1357)	50226(126454)
Mean(SD)	73.08(21.95)	68.42(21.72)	75.63(19.69)	54.84(13.50)	59.94(10.75)	69.69(18.15)	67.26(16.78)	74.20(20.65)	70.48(20.78)
Median(Q1,Q3)	70.30(58.60,84.00)	65.00(55.00,78.00)	69.75(63.00,90.00)	52.25(48.00,60.00)	60.00(52.00,67.00)	67.00(57.00,79.90)	65.00(56.00,76.00)	72.00(60.00,85.00)	67.64(57.04, 80.67)
Reporters									
Consumer	16165(24.66)	17211(32.48)	396(18.88)	20(2.47)	9(0.27)	2998(12.99)	2012(15.29)	203(9.30)	39014(23.91)
Unspecified	141(0.22)	287(0.54)	10(0.48)	0(0.00)	1075(32.19)	69(0.30)	1896(14.41)	38(1.74)	3516(2.15)
Lawyer	32(0.05)	20(0.04)	0(0.00)	0(0.00)	0(0.00)	4(0.02)	0(0.00)	0(0.00)	56(0.03)
Physician	20592(31.42)	23376(44.12)	1241(59.15)	54(6.67)	155(4.64)	16667(72.21)	7663(58.25)	1541(70.62)	71289(43.68)
Pharmacist	16619(25.36)	9770(18.44)	425(20.26)	735(90.85)	2101(62.90)	3032(13.14)	1442(10.96)	305(13.98)	34429(21.10)
Other health professional	11996(18.30)	2320(4.38)	26(1.24)	0(0.00)	0(0.00)	310(1.34)	142(1.08)	95(4.35)	14889(9.12)
Report countries									
USA	28285(43.15)	20007(37.76)	892(42.52)	6(0.74)	0(0.00)	4424(19.17)	2599(19.76)	586(26.86)	56799(34.80)
China	2045(3.12)	1370(2.59)	5(0.24)	802(99.13)	3336(99.88)	1184(5.13)	1612(12.25)	2(0.09)	10356(6.35)
France	5879(8.97)	3348(6.32)	180(8.58)	0(0.00)	0(0.00)	1186(5.14)	867(6.59)	202(9.26)	11662(7.15)
Japan	10431(15.91)	15376(29.02)	122(5.82)	0(0.00)	0(0.00)	6327(27.41)	3833(29.14)	333(15.26)	36422(22.32)
Canada	1732(2.64)	756(1.43)	71(3.38)	0(0.00)	0(0.00)	209(0.91)	743(5.65)	101(4.63)	3612(2.21)
UK	1107(1.69)	782(1.48)	113(5.39)	0(0.00)	0(0.00)	488(2.11)	200(1.52)	145(6.65)	2835(1.74)
Other countries	16066(24.52)	11345(21.40)	715(34.07)	1(0.12)	4(0.12)	8073(40.13)	3301(25.09)	813(37.25)	40318(24.71)
Serious outcomes									
Death	18891(28.82)	11057(20.87)	411(19.59)	21(2.60)	31(0.93)	5946(25.76)	4173(31.72)	558(25.57)	41088(25.18)
Hospitalization-initial/prolonged	25970(39.62)	18986(35.83)	847(40.37)	527(65.14)	1705(51.05)	10014(43.39)	4017(30.54)	801(36.71)	62867(38.52)
Life-threatening	4382(6.69)	3073(5.80)	131(6.24)	87(10.75)	160(4.79)	929(4.03)	1048(7.97)	133(6.10)	9943(6.09)
Disability	1104(1.68)	1495(2.82)	42(2.00)	32(3.96)	154(4.61)	277(1.20)	240(1.82)	28(1.28)	3372(2.07)

During data processing, the unique adverse event report identifier (PRIMARYID) was used to link drug-specific PRIMARYID lists with the main database. When a single report involved multiple ICIs, the record was expanded into multiple rows using a one-to-many mapping, generating separate entries for each drug. This approach was applied to reduce potential confounding related to combination therapy and to enable independent assessment of reporting patterns for individual drugs, thereby improving the accuracy and interpretability of the regression analyses.

In univariable logistic regression analyses, the occurrence of respiratory adverse events (0 = no event, 1 = event) was defined as the dependent variable, with age, sex, body weight, reporting country, and drug class entered individually as independent variables. Odds ratios (ORs), 95% confidence intervals (CIs), and corresponding P values were estimated.

For multivariable analyses, variables showing statistical significance in univariable analyses (P < 0.05), along with other clinically relevant covariates with potential confounding effects (including sex, age, reporting country, and drug class), were entered simultaneously into multivariable logistic regression analyses. Associations with respiratory adverse events were evaluated after adjustment. Results are presented as ORs with 95% CIs and corresponding P values and are displayed using forest plots.

All statistical analyses were performed using R software (version 4.5.1). Logistic regression analyses were conducted with the glm function, parameter estimation and significance testing were carried out using the broom package, and forest plots were generated with the ggplot2 package.

## 3 Results

### 3.1 Baseline demographic characteristics

**Table 1** presents the characteristics of adverse event (AE) reports associated with eight PD-1/PD-L1 inhibitors in the FAERS database. In total, 163,193 individual cases were identified, corresponding to 345,150 reported AEs. Reports related to PD-1 inhibitors—nivolumab, pembrolizumab, cemiplimab, toripalimab, and tislelizumab—were more frequent than those involving PD-L1 inhibitors (atezolizumab, durvalumab, and avelumab), accounting for approximately 76.5% and 23.5% of cases, respectively. Within the PD-1 inhibitor group, nivolumab and pembrolizumab together contributed over 70% of all reports, consistent with their broad clinical use. Among PD-L1 inhibitors, atezolizumab predominated, representing more than two-thirds of reports in this subgroup.

Across all AE reports, cases with serious outcomes, exceeding 80% for most ICIs. The proportion of serious events was particularly high for toripalimab (99.6%) and tislelizumab (99.9%). Among serious outcomes, hospitalization was the most frequently reported event, with proportions ranging from approximately 30% to 65% across agents. Death ranked second and showed substantial variability; durvalumab had the highest proportion of fatal outcomes (31.7%), whereas tislelizumab and toripalimab showed the lowest proportions (<3%). Although report volumes differed markedly among drugs, which may introduce reporting bias, these data suggest that AEs associated with ICIs are frequently clinically serious and merit careful attention.

Regarding demographic characteristics, male patients accounted for a larger proportion of reports, particularly for nivolumab (56.4%) and pembrolizumab (49.3%). For some agents, including toripalimab and tislelizumab, sex information was frequently missing, reflecting incomplete reporting. Age was mainly distributed in the 45–64 and ≥65 year groups, with older adults (≥65 years) forming the largest subgroup, indicating that ICI-related adverse events were more commonly reported among middle-aged and elderly patients.

Physicians were the main reporting source for most drugs, contributing 44.1% of pembrolizumab reports and 31.4% of nivolumab reports, followed by pharmacists and consumers. In contrast, pharmacist-submitted reports accounted for a notably high proportion for toripalimab and tislelizumab (90.85% and 62.90%, respectively), which may reflect differences in pharmacovigilance practices across healthcare systems.

Geographically, the United States and Japan were the principal sources of reports. For nivolumab and pembrolizumab, 43.15% and 37.76% of reports originated from the United States, whereas higher proportions of reports for atezolizumab and durvalumab were submitted from Japan (27.4% and 29.1%). Reports from China were almost exclusively associated with domestically developed agents, namely toripalimab (99.13%) and tislelizumab (99.88%), highlighting the strong influence of regional drug approval on reporting patterns.

### 3.2 Signal strength of respiratory adverse events

**Figure 2:**
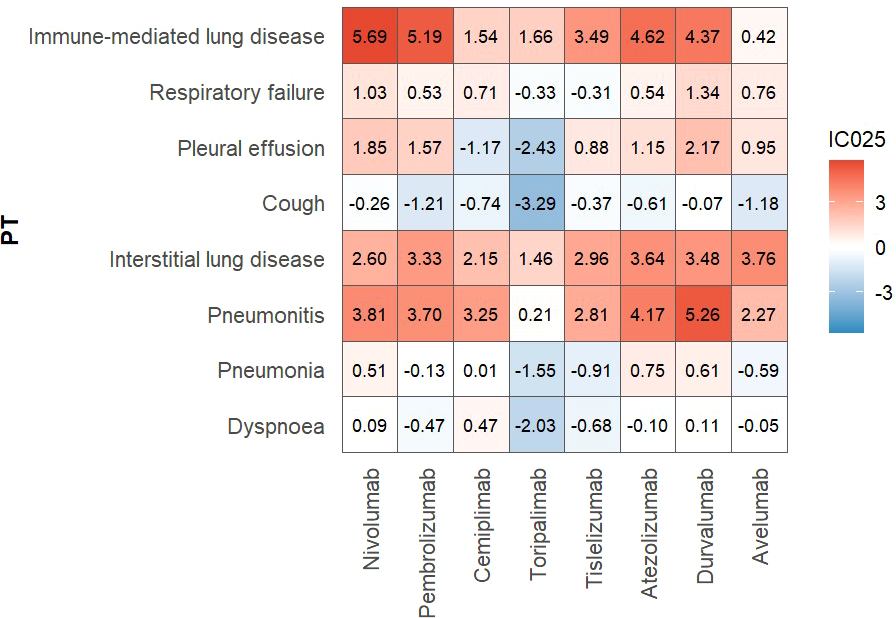
Heatmap of top 8 respiratory PTs.

Among the eight PD-1/PD-L1 inhibitors, eight frequent respiratory adverse events (encompassing both non-specific symptoms and specific pirAEs with high reporting frequency and clinical relevance were selected for further analysis. Using the lower limit of the information component (IC_025_) as the signal detection threshold, a heatmap was generated to visualize the association between individual drugs and pulmonary adverse events at the PT level in **Figure 2**. The heatmap revealed that certain PD-1/PD-L1 inhibitors exhibited relatively strong signals for specific pulmonary adverse event PTs, suggesting potential drug-specific risk patterns. Detailed quantitative results are presented in **Table 2**. At the SOC level, the proportion of adverse events categorized as “Respiratory, thoracic and mediastinal disorders” varied considerably across the eight agents. Ranked from highest to lowest, the proportions were observed for durvalumab (10.64%), avelumab (7.90%), atezolizumab (7.43%), nivolumab (7.09%), cemiplimab (6.77%), pembrolizumab (6.59%), tislelizumab (4.63%), and toripalimab (3.06%). It was excluded for the sake of clarity in the table presentation.

**Table 2:** Signal detection of the most frequent respiratory AEs.

No.	PT	N	ROR(95%CI)	PRR(χ2)	EBGM(95%CI)	IC_025_	positive signal
Nivolumab							
1	Dyspnoea	1921	1.11(1.06-1.16)	1.11(22.76)	1.11(1.06-1.16)	0.09	
2	Pneumonia	1792	1.50(1.43-1.57)	1.49(292.85)	1.49(1.42-1.56)	0.51	
3	Pneumonitis	1318	15.76(14.91-16.66)	15.65(17257.61)	14.98(14.17-15.83)	3.81	Y
4	Interstitial lung disease	876	6.66(6.23-7.12)	6.64(4113.75)	6.52(6.10-6.98)	2.60	Y
5	Cough	839	0.90(0.84-0.96)	0.89(10.49)	0.89(0.84-0.96)	-0.26	
6	Pleural effusion	722	3.96(3.67-4.26)	3.94(1568.74)	3.91(3.63-4.21)	1.85	Y
7	Respiratory failure	595	2.24(2.07-2.43)	2.23(403.75)	2.23(2.05-2.41)	1.03	Y
8	immune-mediated lung disease	282	95.79(83.88-109.40)	95.64(20409.03)	74.14(64.91-84.67)	5.69	Y
Pembrolizumab							
1	Interstitial lung disease	1283	11.13(10.53-11.77)	11.05(11389.53)	10.75(10.17-11.37)	3.33	Y
2	Dyspnoea	1170	0.76(0.72-0.81)	0.76(86.72)	0.76(0.72-0.81)	-0.47	
3	Pneumonitis	1093	14.59(13.73-15.50)	14.49(13213.88)	13.98(13.16-14.85)	3.70	Y
4	Pneumonia	1037	0.97(0.91-1.03)	0.97(0.91)	0.97(0.91-1.03)	-0.13	
5	Pleural effusion	534	3.28(3.01-3.57)	3.27(836.12)	3.25(2.99-3.54)	1.57	Y
6	Respiratory failure	381	1.61(1.45-1.78)	1.61(87.05)	1.60(1.45-1.77)	0.53	
7	Cough	397	0.47(0.43-0.52)	0.47(230.36)	0.48(0.43-0.53)	-1.21	
8	immune-mediated lung disease	186	64.62(55.29-75.53)	64.55(9892.74)	55.02(47.08-64.31)	5.19	Y
Cemiplimab							
1	Dyspnoea	83	1.40(1.13-1.74)	1.40(9.40)	1.40(1.12-1.73)	0.47	
2	Pneumonia	55	1.33(1.02-1.73)	1.33(4.46)	1.33(1.02-1.73)	0.01	
3	Pneumonitis	50	16.63(12.59-21.97)	16.50(727.22)	16.47(12.47-21.77)	3.25	Y
4	Interstitial lung disease	34	7.38(5.27-10.34)	7.34(186.41)	7.34(5.24-10.29)	2.15	Y
5	Cough	28	0.86(0.60-1.25)	0.87(0.59)	0.87(0.60-1.25)	-0.74	
6	Respiratory failure	24	2.61(1.75-3.90)	2.60(23.74)	2.60(1.74-3.89)	0.71	Y
7	Pleural effusion	6	0.94(0.42-2.10)	0.94(0.02)	0.94(0.42-2.10)	-1.17	
8	immune-mediated lung disease	6	45.99(20.61-102.61)	45.94(262.53)	45.73(20.50-102.02)	1.54	Y
Toripalimab							
1	Interstitial lung disease	10	8.56(4.60-15.95)	8.51(66.35)	8.51(4.57-15.85)	1.46	Y
2	Dyspnoea	7	0.46(0.22-0.97)	0.46(4.38)	0.46(0.22-0.97)	-2.03	
3	Pneumonia	7	0.66(0.32-1.40)	0.67(1.18)	0.67(0.32-1.40)	-1.55	
4	Immune-mediated lung disease	6	181.84(81.41-406.17)	181.13(1069.64)	180.26(80.70-402.65)	1.66	Y
5	Respiratory failure	5	2.14(0.89-5.15)	2.14(3.03)	2.14(0.89-5.14)	-0.33	
6	Pneumonitis	4	5.21(1.95-13.90)	5.20(13.56)	5.20(1.95-13.86)	0.21	Y
7	Cough	2	0.24(0.06-0.97)	0.24(4.72)	0.24(0.06-0.98)	-3.29	
8	Pleural effusion	1	0.62(0.09-4.39)	0.62(0.24)	0.62(0.09-4.39)	-2.43	
Tislelizumab							
1	Interstitial lung disease	56	12.26(9.42-15.95)	12.16(573.07)	12.14(9.33-15.80)	2.96	Y
2	Pneumonitis	38	12.66(9.20-17.42)	12.59(405.04)	12.57(9.14-17.30)	2.81	Y
3	Dyspnoea	49	0.83(0.62-1.09)	0.83(1.78)	0.83(0.62-1.10)	-0.68	
4	Cough	35	1.09(0.78-1.52)	1.08(0.24)	1.09(0.78-1.51)	-0.37	
5	Pneumonia	31	0.75(0.53-1.07)	0.75(2.58)	0.75(0.53-1.07)	-0.91	
6	Immune-mediated lung disease	19	148.11(94.08-233.18)	147.65(2725.14)	145.40(92.36-228.92)	3.49	Y
7	Pleural effusion	20	3.16(2.04-4.90)	3.15(29.41)	3.15(2.03-4.89)	0.88	Y
8	Respiratory failure	13	1.42(0.82-2.44)	1.42(1.59)	1.42(0.82-2.44)	-0.31	
9	Chest discomfort	54	5.53(4.23-7.23)	5.49(198.60)	5.49(4.20-7.18)	1.95	Y
Atezolizumab							
1	Pneumonia	721	1.82(1.70-1.96)	1.81(264.74)	1.81(1.68-1.95)	0.75	
2	Interstitial lung disease	613	14.11(13.03-15.29)	13.98(7287.23)	13.79(12.73-14.95)	3.64	Y
3	Pneumonitis	588	20.79(19.15-22.57)	20.59(10741.61)	20.19(18.60-21.92)	4.17	Y
4	Dyspnoea	577	1.01(0.93-1.10)	1.01(0.07)	1.01(0.93-1.10)	-0.10	
5	Immune-mediated lung disease	70	58.93(46.30-75.01)	58.86(3756.89)	55.60(43.68-70.77)	4.62	Y
6	Pleural effusion	160	2.63(2.25-3.07)	2.62(160.11)	2.62(2.24-3.06)	1.15	Y
7	Respiratory failure	152	1.72(1.47-2.02)	1.72(45.75)	1.72(1.47-2.01)	0.54	
8	Cough	232	0.75(0.66-0.85)	0.75(19.93)	0.75(0.66-0.85)	-0.61	
Durvalumab							
1	Radiation pneumonitis	749	1281.90(1169.69-1404.88)	1247.54(580025.1987)	775.99(708.07-850.43)	8.45	Y
2	Pneumonitis	624	46.58(42.99-50.47)	45.56(26619.44)	44.59(41.16-48.32)	5.26	Y
3	Dyspnoea	330	1.21(1.08-1.34)	1.20(11.47)	1.20(1.08-1.34)	0.11	
4	Pneumonia	326	1.71(1.54-1.91)	1.71(95.89)	1.71(1.53-1.90)	0.61	
5	Interstitial lung disease	280	13.31(11.83-14.98)	13.19(3137.03)	13.11(11.65-14.75)	3.48	Y
6	Cough	166	1.11(0.96-1.30)	1.11(1.92)	1.11(0.96-1.30)	-0.07	
7	Pleural effusion	159	5.45(4.66-6.37)	5.42(572.66)	5.41(4.63-6.33)	2.17	Y
8	Lung disorder	137	6.34(5.36-7.50)	6.31(611.18)	6.30(5.32-7.45)	2.35	Y
9	Respiratory failure	130	3.07(2.58-3.65)	3.06(180.46)	3.06(2.57-3.63)	1.34	Y
10	Pulmonary toxicity	98	33.29(27.26-40.66)	33.18(3010.16)	32.67(26.75-39.90)	4.34	Y
11	Immune-mediated lung disease	44	75.49(55.86-102.01)	75.37(3114.38)	72.73(53.82-98.28)	4.37	Y
Avelumab							
1	Interstitial lung disease	76	22.07(17.59-27.69)	21.71(1500.57)	21.68(17.28-27.20)	3.76	Y
2	Dyspnoea	57	1.27(0.98-1.65)	1.27(3.22)	1.27(0.98-1.64)	-0.05	
3	Pneumonia	30	0.96(0.67-1.37)	0.96(0.06)	0.96(0.67-1.37)	-0.59	
4	Pneumonitis	23	10.07(6.68-15.16)	10.02(186.69)	10.01(6.65-15.09)	2.27	Y
5	Pulmonary embolism	23	3.20(2.13-4.83)	3.19(34.67)	3.19(2.11-4.81)	0.96	Y
6	Respiratory failure	20	2.87(1.85-4.46)	2.87(24.34)	2.87(1.85-4.45)	0.76	Y
7	Pleural effusion	17	3.53(2.20-5.69)	3.53(30.77)	3.52(2.19-5.67)	0.95	Y
8	Cough	17	0.69(0.43-1.12)	0.69(2.30)	0.69(0.43-1.12)	-1.18	
9	Immune-mediated lung disease	3	30.30(9.75-94.10)	30.28(84.73)	30.21(9.73-93.82)	0.42	Y

Based on the predefined criterion requiring simultaneous positivity across four signal detection methods (ROR, PRR, EBGM, and IC_025_), the signal strength results are summarized in **Table 2**. Among the eight immune checkpoint inhibitors, Within this respiratory AE profile, classic pirAEs such as immune-mediated lung disease, interstitial lung disease (ILD), and pneumonitis consistently demonstrated prominent drug-related signals, exhibiting a striking similarity in high-signal patterns across agents. This convergence suggests a shared tendency of PD-1/PD-L1 inhibitors to disrupt pulmonary immune homeostasis.

Beyond the overall signal patterns shared across PD-1/PD-L1 inhibitors, several ICIs exhibited drug-specific respiratory adverse event profiles that differed from those observed with other agents. Tislelizumab, for example, showed a pronounced signal for chest discomfort, with 54 reported cases (0.90%) and relatively strong signal strength (ROR 5.53, 95% CI 4.23–7.23; IC_025_ 1.95), suggesting a more concentrated distribution of chest-related adverse events. Durvalumab demonstrated a particularly strong association with radiation-related lung injury. Radiation pneumonitis accounted for 749 reports (SOC proportion 2.68%) and showed an exceptionally high signal intensity (ROR 1281.90, 95% CI 1169.69–1404.88; IC_025_ 8.45). This finding highlights the importance of careful risk–benefit assessment, especially given the established survival benefit of durvalumab as consolidation therapy after concurrent chemoradiotherapy in patients with stage III non–small cell lung cancer (19). In addition, several other respiratory adverse events showed elevated signal intensities for durvalumab, including respiratory failure (130 cases; ROR 3.07; IC_025_ 1.34), pulmonary toxicity (98 cases; ROR 33.29; IC_025_ 4.34), and pleural effusion (159 cases; ROR 5.45; IC_025_ 2.17). This signal profile is consistent with its clinical use context, in which prior radiation-related lung injury combined with immune activation may increase susceptibility to radiation pneumonitis and related pulmonary toxicities, with progression to severe outcomes in a subset of patients (20, 21). For avelumab, pulmonary embolism emerged as a distinct and disproportionately elevated signal (23 cases; ROR 3.20; IC_025_ 0.96). Respiratory failure (20 cases; ROR 2.87; IC_025_ 0.76) and pleural effusion (17 cases; ROR 3.53; IC_025_ 0.95) also showed relatively increased signal levels. These findings are in line with previous observations suggesting that ICIs may be associated with thromboembolic events, potentially through immune-mediated vascular injury, thereby contributing to secondary pulmonary complications (22).

Overall, PD-1 inhibitors showed higher signal intensities than PD-L1 inhibitors, with nivolumab and pembrolizumab ranking highest in both the number of reported cases and signal strength. This pattern may be partly explained by their earlier approval, broader clinical indications, and longer duration of use, which together have led to larger cumulative reporting volumes and greater sensitivity for signal detection. These observations underscore the need for careful clinical monitoring during ICI therapy, particularly for the early identification and management of inflammatory pulmonary events, to reduce the risk of progression to interstitial lung disease or severe pulmonary dysfunction.

### 3.3 Treatment duration and temporal trends

**Figure 3:**
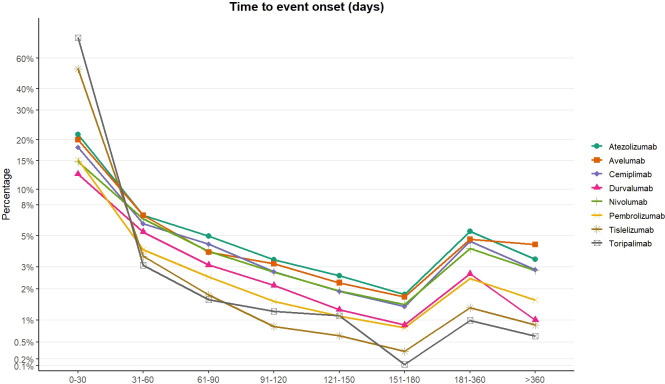
Time to onset of adverse events by drug.

**Figure 3** depicts the temporal distribution of adverse event onset across different ICIs. A broadly consistent pattern was observed across agents, characterized by an early peak after treatment initiation, followed by a decline and a modest increase with longer exposure. The ≤30-day interval represented the period with the highest concentration of reported events, accounting for 14.77% of cases for nivolumab, 15.19% for pembrolizumab, 18.02% for cemiplimab, 21.56% for atezolizumab, 12.43% for durvalumab, and 20.03% for avelumab. Among domestically developed PD-1 inhibitors, early-onset events were particularly prominent, reaching 78.49% for toripalimab and 51.98% for tislelizumab. Across subsequent intervals between 31 and 180 days, the proportion of reported adverse events generally declined. For example, the proportion for nivolumab decreased from 6.49% to 1.45%, for pembrolizumab from 4.00% to 0.81%, and for atezolizumab from 6.84% to 1.79%. During the 181–360-day window, a secondary increase in reporting was observed for most agents, including nivolumab (4.09%), pembrolizumab (2.44%), cemiplimab (4.58%), atezolizumab (5.37%), durvalumab (2.65%), and avelumab (4.72%). A measurable proportion of late-onset events also persisted beyond 360 days, such as 2.81% for nivolumab, 3.41% for atezolizumab, and 4.35% for avelumab, suggesting that additional risk remains during prolonged therapy. It should be noted that a relatively high proportion of reports contained missing or implausible onset-time information for certain agents, including pembrolizumab (70.79%), durvalumab (71.22%), and nivolumab (61.85%). Although this limitation may affect the precision of temporal estimates, it does not alter the overall observed pattern of early predominance with a subsequent increase after approximately six months. These findings indicate that intensified monitoring should be implemented at the initiation of immunotherapy, while continued vigilance is also warranted during long-term treatment, particularly in the context of sustained immune activation and the potential additive effects of prior or concurrent chemoradiotherapy–related radiation lung injury.

**Figure 4:**
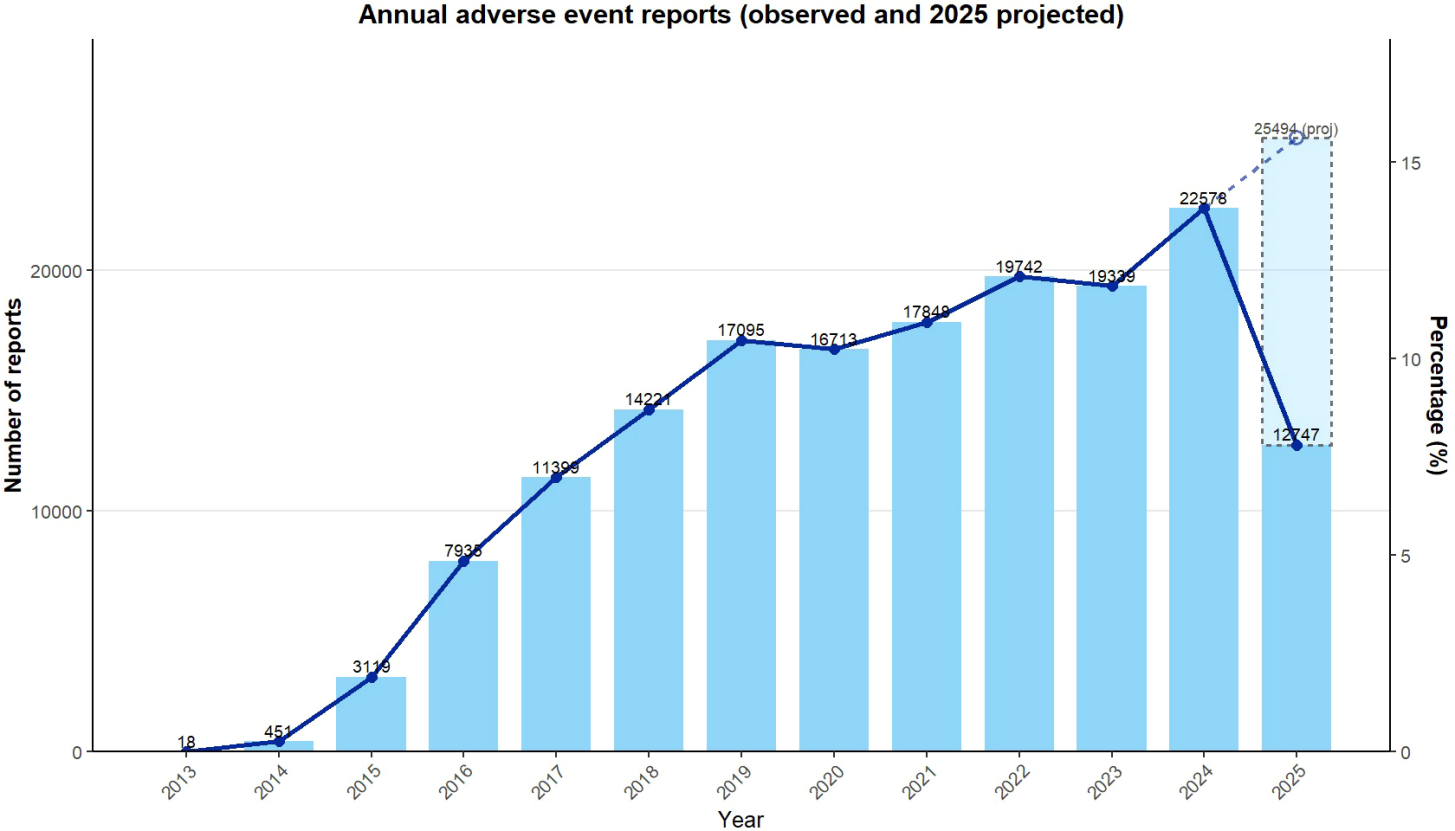
Annual adverse events reports.

Although the eight PD-1/PD-L1 inhibitors differed in both approval timing and the absolute number of reported adverse events, a clear upward trajectory in adverse event reporting was evident over the study period **Figure 4**. This rise appears to be consistent with typical post-marketing patterns of immune checkpoint inhibitors. As clinical use expands, indications broaden, and real-world experience accumulates, the volume of reported adverse events tends to increase gradually. Several factors may have contributed to this sustained growth. Pharmacovigilance systems have continued to mature, and clinicians are now more familiar with immune-related adverse events, which could encourage reporting. At the same time, longer patient survival and extended treatment duration mean that patients remain exposed to these agents for longer periods, increasing the opportunity for adverse events to be observed and documented.

The dataset used in this analysis includes reports up to the second quarter of 2025. For the purpose of displaying annual trends, the value for 2025 was estimated by doubling the number of reports recorded in the first two quarters. This projected component is shown as a dashed bar in **Figure 4**. Accordingly, the 2025 figure should be viewed as illustrative of the overall direction rather than as a precise annual total. The persistent increase in reported adverse events highlights the importance of continued monitoring of the safety profiles of PD-1/PD-L1 inhibitors. Placing these findings within a temporal context may help inform how pharmacovigilance efforts are prioritized and where long-term safety surveillance should remain focused.

### 3.4 Univariable and multivariable analyses

**Figure 5:**
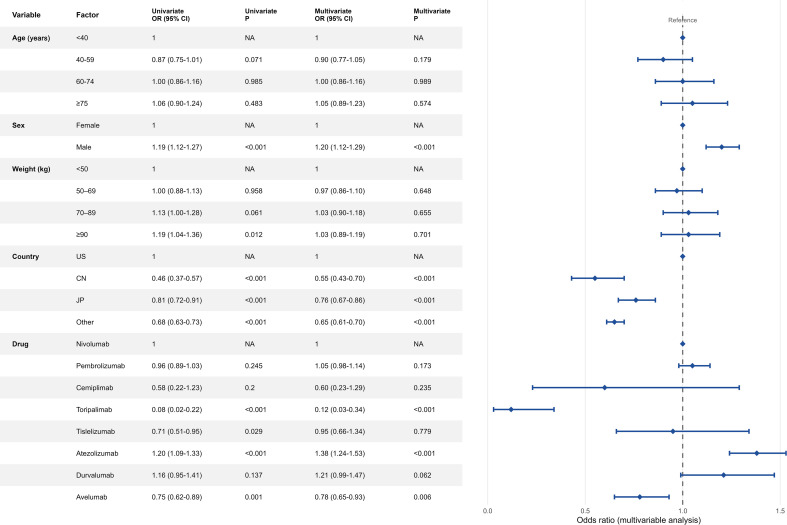
Univariate and multivariate analyses of risk factors.

**Figure 5** summarizes the results of the univariable and multivariable regression analyses. In univariable analyses, sex, reporting country, and drug category were significantly associated with the occurrence of ICI-related adverse events. After adjustment in multivariable logistic regression analyses, these associations for sex, reporting country, and drug type remained statistically significant (P < 0.05), whereas age and body weight categories did not retain statistical significance.

According to the multivariable regression analysis, male patients had a higher reporting risk of adverse events than female patients (OR = 1.20, 95% CI 1.12–1.29, P < 0.001). This difference may be related to sex-specific variations in immune responses or treatment patterns, although the underlying mechanisms remain unclear. After adjustment, neither age nor body weight categories were significantly associated with adverse event occurrence. The modest increase in risk observed with higher body weight in the univariable analysis was attenuated in the multivariable model, suggesting potential confounding by factors such as comorbid conditions or treatment characteristics. Reporting risk also differed by country. Compared with the United States, reports from China were associated with a significantly lower reporting risk (OR = 0.55, 95% CI 0.43–0.70, P < 0.001), while Japan and other countries similarly showed reduced reporting odds (OR = 0.76 and 0.65, respectively; both P < 0.001). These differences may reflect variation in prescribing practices, pharmacovigilance systems, or population characteristics. The influence of country-specific reporting infrastructures should therefore be considered when interpreting these findings. Substantial heterogeneity was observed across drug classes. Using nivolumab as the reference, pembrolizumab, cemiplimab, and tislelizumab did not show statistically significant differences in adjusted analyses, indicating broadly comparable risk profiles. The limited number of cemiplimab reports, however, resulted in wide confidence intervals and warrants further evaluation. In contrast, atezolizumab was associated with a higher reporting risk (OR = 1.38, 95% CI 1.24–1.53, P < 0.001), whereas avelumab showed a lower risk (OR = 0.78, 95% CI 0.65–0.93, P = 0.006). Toripalimab exhibited a markedly reduced odds ratio (OR = 0.12, 95% CI 0.03–0.34, P < 0.001); however, given its recent approval, restricted geographic use, and relatively small number of reports, this result is more likely influenced by exposure size and reporting practices rather than reflecting an intrinsically lower risk.

## 4 Discussion

In this study, respiratory adverse events associated with PD-1/PD-L1 inhibitors were systematically evaluated using data from the FAERS, with a focus on characterizing pirAEs and identifying relevant safety signals. The results indicate that pirAEs constitute a clinically severe and potentially fatal category of ICI-related toxicities, most commonly presenting as immune-related pneumonitis, interstitial lung disease, and respiratory failure. Previous reports have described mortality rates of up to 20% for ICI-associated pneumonitis, highlighting its clinical significance and the need for heightened awareness in practice. Compared with earlier studies, the present analysis captures long-term reporting trends from 2004 to 2025 across eight PD-1/PD-L1 inhibitors and applies four internationally recognized disproportionality methods (ROR, PRR, IC, and EBGM). Safety signals were defined using a conservative criterion requiring concurrent positivity across all four metrics, which strengthens the reliability of signal detection (23). In addition, multivariable logistic regression analyses were performed with adjustment for key confounders, including age, sex, and reporting country, allowing the identification of independent factors associated with pirAEs and providing more detailed insight into risk heterogeneity across populations and drug classes. Although confirmation in prospective studies is still needed, these findings offer clinically relevant evidence to support ongoing pharmacovigilance efforts and early risk identification.

Overall, signal intensities were generally stronger for PD-1 inhibitors than for PD-L1 inhibitors, which may reflect more pronounced T-cell activation and cellular immune enhancement associated with PD-1 blockade, thereby increasing susceptibility to immune-mediated inflammatory injury in lung tissue. Specifically, pneumonitis, interstitial lung disease, and immune-mediated lung disease showed apparent overlap in signal strength and clinical features. Pneumonitis, defined as a PT distinct from infectious pneumonia, refers to noninfectious inflammatory involvement of the lung parenchyma and interstitium, typically related to immune activation or drug exposure. In the setting of immunotherapy, pneumonitis is often regarded as one of the earlier and more sensitive immune-related manifestations, frequently arising during the initial phase of treatment and presenting with acute inflammatory changes or diffuse radiographic abnormalities. In the present analysis, pneumonitis generally demonstrated intermediate to high signal strength, suggesting that it may represent an early and relatively common manifestation of immune-mediated pulmonary injury. Interstitial lung disease, by contrast, reflects a more advanced stage characterized by progression from inflammation toward structural lung involvement. Signals for interstitial lung disease were consistently detected across agents, indicating a degree of persistence and class-wide similarity, and suggesting that ICIs may be associated with sustained interstitial inflammatory processes during immune activation. Immune-mediated lung disease, although less frequently reported, exhibited the strongest overall signal intensity, with ROR and IC025 values ranking highest among the pulmonary adverse events analyzed. This pattern is consistent with a higher degree of drug specificity and immune dependence. Clinically, such events are often linked to pronounced immune activation, extensive pulmonary inflammation, and prolonged immune dysregulation, and may be more likely to present with severe respiratory manifestations, including respiratory failure.

In addition, although the domestically developed PD-1 inhibitors toripalimab and tislelizumab were associated with relatively smaller overall numbers of adverse event reports, both agents demonstrated disproportionately high signal strength for immune-mediated lung disease and interstitial lung disease. This finding suggests a potential risk of immune-mediated interstitial lung injury. However, given that the clinical use of these agents remains relatively concentrated and that most reports originate from a single geographic region, the limited sample size may lead to overestimation of signal metrics. Furthermore, regional differences in reporting practices and pharmacovigilance systems may introduce additional bias. Therefore, the signal strength associated with these agents should be interpreted with caution.

Regarding the timing of onset, most adverse events in this study occurred within 60 days after treatment initiation, indicating a predominantly early-onset pattern. This finding is consistent with the analysis by Li et al (24), based on combined FAERS and VigiBase data. In their study, they specifically demonstrated that over 50% of pulmonary immune-related adverse events manifested within the initial 60 days of therapy. Highlighted that events occurring within this early window were associated with a higher mortality rate, underscoring the critical need for prompt clinical vigilance. Similarly, a large multicenter study involving 20 institutions reported that most immune-related adverse events developed within four months after initiation of anti–PD-1 therapy (25), with a median onset time of 3.9 months (interquartile range [IQR], 2.1–7.3 months) (26). Earlier FAERS-based studies have described an even shorter median onset for PD-1–associated pneumonitis (28 days; IQR, 12–84.25 days), which broadly aligns with our observations (27). Importantly, although early-onset events predominated, pirAEs were not restricted to the initial treatment phase. Owen et al. (25). reported that late-onset immune-related toxicities (onset >12 months), including pneumonitis, may occur or recur during prolonged immunotherapy. Together, these findings point to a biphasic temporal pattern of immune-related toxicity, with an early vulnerability period after treatment initiation and a later phase potentially related to cumulative exposure or immune dysregulation. Consistent with this concept, Zhang et al (28). also noted that immune-related pneumonitis tends to occur early, followed by delayed presentations, which parallels the “early predominance with late resurgence” pattern observed in our analysis (29). Given the severity of pneumonitis among ICI-related toxicities, these data support a monitoring strategy that emphasizes intensified surveillance during the first 3–6 months of treatment, alongside continued vigilance during long-term therapy and after treatment discontinuation.

Because ICIs are most commonly prescribed to older patients (30, 31), irAEs in elderly populations account for a substantial proportion of reported cases. Prior studies have shown that the distribution of irAEs across organ systems varies considerably with age. Cardiovascular irAEs tend to peak in patients aged 75–84 years, whereas endocrine irAEs generally decline with advancing age. In contrast, hepatobiliary, gastrointestinal, and ocular irAEs become less frequent in older age groups, while renal and musculoskeletal irAEs increase, indicating differential age-related vulnerability across organ systems. Although the proportion of severe adverse events appears to decrease slightly among patients aged ≥85 years, mortality risk continues to rise with increasing age. These observations are consistent with our findings, in which pirAEs were reported less frequently overall but were associated with a disproportionately high fatality once they occurred. Experimental evidence suggests that aging may enhance anti–PD-(L)1–related pulmonary toxicity through age-related alterations in the immune microenvironment (32). In parallel, multicenter clinical studies have reported significantly higher mortality among elderly patients who develop severe irAEs, such as pneumonitis, compared with younger patients (33). Reduced organ reserve and a higher burden of comorbidities in older individuals may further exacerbate the risk of adverse outcomes (34). Indeed, several studies using the Charlson Comorbidity Index have demonstrated that higher comorbidity burden is associated with poorer clinical outcomes (35). Taken together, these findings highlight the need for careful management of elderly patients receiving ICIs. A comprehensive evaluation of functional status, comorbid conditions, and expected therapeutic benefit may help guide individualized monitoring and supportive care strategies, with the aim of reducing the risk of severe or fatal pirAEs.

In several single-agent analyses, we observed strong safety signals, among which pulmonary embolism (PE) exhibited a particularly prominent association (ROR ≈ 3.20, 95% CI 2.11–4.81), suggesting that PE may represent a serious adverse event that warrants attention during ICI therapy. At present, direct evidence linking avelumab specifically to PE remains limited; however, our findings are consistent with existing evidence indicating an overall increased risk of thrombotic events associated with ICIs. Previous studies have shown that PD-1/PD-L1 inhibitors are associated with an elevated risk of venous thromboembolism (VTE), including severe manifestations such as PE (36, 37). In a large retrospective analysis, the incidence of VTE among patients receiving ICIs reached up to 24%, and VTE occurrence was associated with reduced overall survival (hazard ratio [HR] = 1.22(22)). From a mechanistic perspective, immune activation induced by ICIs may contribute to a prothrombotic milieu through systemic inflammation, endothelial injury, and increased release of procoagulant mediators (38). Tumor-related factors, including cancer type, tumor burden, baseline inflammatory status, and the activity of myeloid-derived suppressor cells, may further influence thrombotic susceptibility in this setting.

Our analysis also identified an association between durvalumab and radiation pneumonitis (RP), which aligns with evidence from both clinical trials and real-world studies. In the PACIFIC trial, the incidence of grade ≥3 RP was comparable between the durvalumab and placebo groups (3.1% vs. 2.6%), with no statistically significant difference (39). However, growing real-world evidence indicates that the overall risk of RP—particularly low- to moderate-grade pneumonitis (grade ≥2)—may be higher among patients receiving durvalumab as consolidation therapy following concurrent chemoradiotherapy (CRT). A large cohort study reported that the incidence of grade ≥2 pneumonitis after durvalumab reached 22.1%, significantly exceeding that observed with CRT alone (13.9%, P < 0.001), and further demonstrated an increased risk of grade 2 pneumonitis associated with durvalumab use (HR = 1.45, 95% CI 1.09–1.93) (19). Radiotherapy-related dosimetric factors, such as lung V20 >30% and mean lung dose (MLD) >17 Gy, have also been recognized as key contributors to RP risk, underscoring the importance of radiotherapy planning (40). Taken together, careful patient selection, optimization of radiation dose distribution, and closer attention to early radiographic changes and inflammatory management may help reduce pneumonitis risk and support treatment adherence and clinical outcomes in patients treated with durvalumab.

In contrast to our findings, Ladjevardi et al. reported that pulmonary irAEs were numerically more frequent in the PD-L1 inhibitor group, with the overall toxicity profile suggesting a more favorable safety pattern for PD-L1 inhibitors compared with PD-1 inhibitors (41). A meta-analysis evaluating immunotherapy combined with chemotherapy likewise found a higher risk of grade 1–2 pneumonitis associated with PD-L1 inhibitors than with PD-1 inhibitors (42). Taken together, these apparently divergent results suggest that PD-1 inhibitors are not invariably associated with more severe pulmonary toxicity and that the influence of drug class on toxicity profiles is inherently complex. Such complexity likely reflects interactions among multiple factors, including tumor type, line of therapy, and treatment strategy, particularly whether immune checkpoint inhibitors are administered as monotherapy or in combination with chemotherapy or radiotherapy. The present study focused exclusively on PD-1/PD-L1 inhibitor monotherapy, whereas combination regimens are increasingly used in routine clinical practice. This difference in treatment context may partly explain the inconsistency between our findings and those of previous reports. Lin et al. reported a higher risk of pulmonary embolism (PE) with PD-1 inhibitors compared with PD-L1 inhibitors in a meta-analysis, which does not fully align with our observation that PE emerged as a distinct and unusually prominent signal for avelumab (43). Differences in study design, patient populations, and outcome definitions may account for this discrepancy, indicating that further external validation is required before definitive conclusions can be drawn. Multicenter data have also shown that late-onset irAEs occurring at 6–12 months and beyond one year are not uncommon, underscoring the need to incorporate long-term surveillance into routine follow-up of patients receiving immune checkpoint inhibitors (44).

This study provides a systematic evaluation of ICI-associated pulmonary adverse events using the FAERS database; nevertheless, several limitations intrinsic to spontaneous reporting systems should be considered. Report quality in FAERS is heterogeneous and often affected by missing information, duplicate or redundant submissions, and incomplete capture of off-label use beyond FDA-approved indications. In addition, the database lacks detailed clinical data, including treatment regimens, concomitant therapies, treatment duration, and imaging findings, which limits accurate incidence estimation and precludes causal inference. Potential misclassification may also arise from MedDRA coding practices, particularly for pulmonary conditions with overlapping clinical phenotypes, such as pneumonitis, infectious pneumonia, and interstitial lung disease. Although deduplication and data standardization were performed, inconsistencies related to MedDRA version updates cannot be entirely excluded. Based on our data preprocessing, no major changes in MedDRA terminology relevant to this analysis were identified during the study period; however, reporting errors and subjective interpretation by reporters remain possible. FAERS further offers limited control over important confounders, including prior radiotherapy, pre-existing lung disease, concurrent infection, or tumor progression, which may be incorrectly attributed to drug exposure. Differences in approved indications and prescribing practices across ICIs may also influence between-drug comparisons. Moreover, disproportionality analyses are intended to detect statistical associations rather than establish causality. Although four complementary signal detection methods were applied to improve robustness, residual confounding is unavoidable. Signal stability is also influenced by differences in approval timing and exposure size, with earlier-approved agents yielding more stable estimates and drugs with smaller reporting volumes being more susceptible to signal inflation. Finally, combination treatment scenarios were not examined in this study, and the toxicity profiles of ICIs used in conjunction with chemotherapy or radiotherapy therefore remain incompletely characterized.

It is important to emphasize that these multivariable regression results reflect statistical associations in adverse event reporting rather than causal relationships. Given the inherent limitations of spontaneous reporting systems, potential residual confounding, and insufficient sample sizes for certain agents, the present analysis should be regarded as exploratory. For example, although the higher reporting risk observed in male patients is consistent with some previous observational studies and may be related to sex-specific differences in immune responses, baseline epidemiology, or smoking habits (45). Similarly, the significant differences in reporting risk across countries more likely reflect variations in drug market approvals, prescribing practices, national pharmacovigilance infrastructures, and population characteristics, rather than true biological differences. The heterogeneity observed across drug classes may also stem from their approved scope of use, rather than reflecting an intrinsically lower biological risk. Therefore, caution must be exercised to avoid over-interpreting the biological plausibility of these findings. Further validation through prospective cohort studies and mechanistic investigations is required.

Several avenues for future research can be considered. Public spontaneous reporting databases, such as FAERS, VigiBase, EudraVigilance, and JADER, differ in reporting quality, regional treatment practices, and coding conventions. Findings based on a single database are therefore prone to systematic bias. Cross-database validation and integrated analyses may help improve signal robustness, particularly when combined with methods such as Bayesian shrinkage or self-controlled case series designs to better account for regional and temporal confounding. Further progress also depends on improved mechanistic insight. Integrating real-world safety data with imaging, pathology, and immunogenomic information may allow more precise characterization of phenotypes and immune signatures underlying PD-1/PD-L1–associated pulmonary toxicity. From a clinical perspective, multicenter prospective cohorts developed in line with standardized guidelines are needed to support risk prediction. Models incorporating baseline imaging features, pulmonary function, prior lung disease, and comorbidity burden could provide a more reproducible basis for treatment selection and follow-up strategies. Drug-specific evaluation remains important. For durvalumab, additional real-world studies are needed to better define the interaction between drug exposure, radiotherapy parameters, and host-related factors in the development of radiation pneumonitis. For avelumab, signals related to thrombotic events and infusion reactions warrant focused investigation of immune–coagulation pathways and identification of susceptible patient subgroups (46). Finally, establishing an evidence-to-practice framework that links signal detection with guideline development, clinical implementation, and outcome assessment may facilitate the translation of pharmacovigilance findings into routine care, particularly for elderly and other high-risk populations.

## 5 Conclusions

This FAERS-based analysis indicates that PD-1/PD-L1 inhibitors are associated with consistent safety signals for respiratory adverse events, particularly immune-related pneumonitis and interstitial lung disease. These events occur mainly during the early phase of treatment, although delayed onset is also observed with prolonged exposure. Elderly patients accounted for a substantial proportion of reported adverse events, highlighting the need for continued clinical vigilance. Taken together, real-world pharmacovigilance data offer valuable evidence to inform risk stratification and support clinical decision-making in immunotherapy.

## Data Availability

The original contributions presented in the study are included in the article/supplementary material. Further inquiries can be directed to the corresponding author.
